# *Notes from the Field:* An Outbreak of West Nile Virus — Arizona, 2019

**DOI:** 10.15585/mmwr.mm7004a4

**Published:** 2021-01-29

**Authors:** Irene Ruberto, Melissa Kretschmer, Karen Zabel, Rebecca Sunenshine, Kirk Smith, John Townsend, Danielle Richard, Laura M. Erhart, Nicholas Staab, Ken Komatsu, Heather Venkat

**Affiliations:** ^1^Arizona Department of Health Services; ^2^Maricopa County Department of Public Health, Phoenix, Arizona; ^3^Career Epidemiology Field Officer Program, Center for Preparedness and Response, CDC; ^4^Vector Control Division, Maricopa County Environmental Services Department, Phoenix, Arizona.

West Nile virus (WNV), a mosquitoborne flavivirus,* was first identified in the United States in 1999 and first reported in Arizona in 2003 (with 12 human cases); 391 human cases were reported in 2004. Since that time, a median of 103 cases (range = 21–391) have been reported in Arizona annually.^†^ During week 28 in 2019, the Arizona Department of Health Services (ADHS) recorded the highest weekly WNV case count (23) ever reported in Arizona (an incidence of 0.32 cases per 100,000 population) (Figure). This prompted ADHS to investigate the outbreak’s severity to inform prevention, resource allocation, and public messaging.

**FIGURE:**
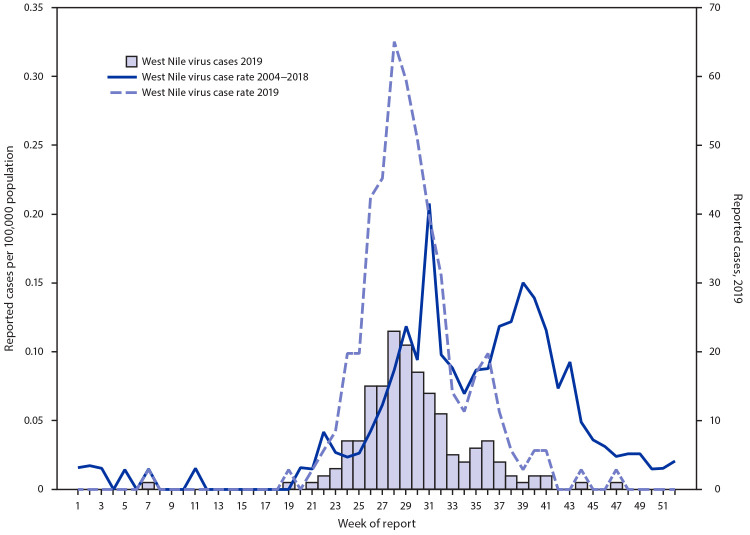
West Nile virus disease cases and incidence per 100,000 population — Arizona, 2004–2019

The 2019 total of 174 WNV cases reported in Arizona was the largest number in the state since 2004, and the second largest number of any state in the United States for that year (*1*). The 2019 incidence in Arizona (2.4 cases per 100,000 population; 65% confirmed) was 50% above the median annual incidence for 2005–2018 (1.6 cases per 100,000 population). Maricopa County, which includes the greater metropolitan Phoenix area, reported 3.7 cases per 100,000 population (the highest county rate since 2004). Cases were classified according to the national WNV surveillance case definition (*2*).

Arizona usually experiences a biphasic WNV season during the summer monsoon rains, with first peak cases occurring in early August and the second peak in late September (*3*). In 2019, the first peak occurred during week 28 (mid-July), 3 weeks earlier than the mean first peak during 2004–2018 (week 31, range = 26–46). The second peak in 2019 occurred during week 35 (end of August), 3 weeks earlier than the mean during 2004–2018 (week 38, range = 36–46). The number of cases during the first peak in 2019 was 72% above the 2004–2018 first peak average, whereas the number of cases during the second peak was 22% below the second peak average for these years.

In 2019, among 174 WNV cases reported across the state, 132 (76%) were identified as neuroinvasive diseases (130 were either meningitis or encephalitis), similar to recent state and national trends (*1*,*4*). Demographics of persons infected in 2019 did not differ significantly from those infected during previous years in Arizona; 58% of cases were in males and the median age was 64 years (range = 6–92 years). The case-fatality rate for all 174 cases was 10%, similar to historical statewide data (median = 7%, range = 0–22%). Statewide, 23 WNV viremic blood donors were reported in 2019, 52% higher than the historical median for available years (2006–2018) (*1*).

In Maricopa County, 0.60% (417 of 69,487) mosquito pools tested positive for WNV in 2019 compared with 0.18% (124 of 67,146) in 2018 and 0.34% (209 of 60,486) in 2017 (*5*). In addition, the vector index^§^ (WNV transmission activity in mosquito populations) (*6*) reached 19.4 in early June 2019, the highest ever detected in the county. This increase in vector index preceded the peak of human WNV cases in Maricopa County by approximately 6 weeks. Arizona experienced a particularly wet fall/winter 2018;^¶^ an increase in vegetation during spring 2019 might have boosted the mosquito and bird populations and amplified WNV sooner than usual, leading to earlier and more human WNV infections (*7*).

ADHS and Maricopa County informed health care providers and the public about the outbreak and distributed educational materials and mosquito repellent across the state. CDC, ADHS, and Maricopa County further investigated WNV presence in the bird and mosquito populations around the Phoenix area.

Ongoing investigation by ADHS, CDC, local vector control agencies, and university partners into factors influencing mosquito abundance, WNV transmission (amplification and suppression), or other factors, such as modeling weather patterns with bird and mosquito population dynamics (*7*), insecticide resistance of mosquitoes, or WNV strain analysis in birds and mosquitoes might help inform future public health prevention and response activities regarding WNV outbreaks.

## Footnotes

*
https://www.cdc.gov/vhf/virus-families/flaviviridae.html

†
https:/www.azdhs.gov/preparedness/epidemiology-disease-control/mosquito-borne/west-nile-virus/index.php#information

§ The vector index is calculated by determining the average number of infected mosquitoes collected per trap in an area, therefore it parallels the mosquito pool positivity.

¶
https://www.weather.gov/psr/Year_in_Review_2018
